# Affective Face Processing Modified by Different Tastes

**DOI:** 10.3389/fpsyg.2021.644704

**Published:** 2021-03-12

**Authors:** Pei Liang, Jiayu Jiang, Jie Chen, Liuqing Wei

**Affiliations:** ^1^Department of Psychology, Faculty of Education, Hubei University, Hubei, China; ^2^Brain and Cognition Research Center (BCRC), Faculty of Education, Hubei University, Hubei, China; ^3^Research Center of Brain and Cognitive Neuroscience, Liaoning Normal University, Liaoning, China; ^4^School of Fundamental Sciences, China Medical University, Shenyang, China

**Keywords:** emotional face, taste, cross-modal, ERP, face search task

## Abstract

Facial emotional recognition is something used often in our daily lives. How does the brain process the face search? Can taste modify such a process? This study employed two tastes (sweet and acidic) to investigate the cross-modal interaction between taste and emotional face recognition. The behavior responses (reaction time and correct response ratios) and the event-related potential (ERP) were applied to analyze the interaction between taste and face processing. Behavior data showed that when detecting a negative target face with a positive face as a distractor, the participants perform the task faster with an acidic taste than with sweet. No interaction effect was observed with correct response ratio analysis. The early (P1, N170) and mid-stage [early posterior negativity (EPN)] components have shown that sweet and acidic tastes modified the ERP components with the affective face search process in the ERP results. No interaction effect was observed in the late-stage (LPP) component. Our data have extended the understanding of the cross-modal mechanism and provided electrophysiological evidence that affective facial processing could be influenced by sweet and acidic tastes.

## Introduction

Facial expressions play a significant role in social situations, and they become even more intriguing during fine dining. So much so that one of the critical social skills for high table culture is detecting others’ emotions while dining. Thus, there is a subtle interplay of cross-modal integration in this matter. How does taste influence facial expression by affecting emotion? More specifically, does the chewing of food influence the affective face identification? These questions motivated our study.

Emotional face detection is indeed a well-established model to study cross-modal sensory integration. Different emotional facial processing has been studied extensively (Schindler and Bublatzky, 2020). Many event-related potential (ERP) studies have shown that there are different ERP components, which involve early (P1, N170), mid-latency (Early Posterior Negativity, EPN), and late (Late Positive Potential, LPP) stages of the emotional facial processing (Xia et al., 2014; Schindler and Bublatzky, 2020). The P1 component represents the early electrocortical processing of facial information, and the emotional modulation has been inconsistent with different experiment tasks (Blechert et al., 2012; Jetha et al., 2012; Sebastian et al., 2019). The N170 component presents the automatic process of the structural encoding of facial expression (Eimer, 2012). EPN implicates the selective attention of emotional information. This component works automatically in some situations as it could be triggered by some emotional background (Junghöfer et al., 2001; Schupp et al., 2006). LPP represents the high-level of cognitive processing and can be modified by top-down attention control (Schupp et al., 2006; Rellecke et al., 2012).

In the real environment, emotional facial recognition can be influenced by other sensory stimuli or background (Ambady and Weisbuch, 2011; Semin and Smith, 2013). The sensory cues from visual, auditory, and olfactory systems have been shown to impact emotional face detection at behavioral and neural levels (Zhou and Chen, 2009; Klasen et al., 2011; Hassin et al., 2013). With incongruent body gestures, for instance, the correct detection ratios of emotional faces decrease and the reaction time increases, compared to those with congruent stimuli (Meeren et al., 2005). When facial information is relatively confusing and challenging to identify the emotion, the additional gesture can provide significant supportive information and help detect facial emotion (van den Stock et al., 2007). Such integration of different visual cues can take place automatically at an early stage, around 100 ms, which is observed even in unattended conditions (Meeren et al., 2005). With additional visual cues, the auditory information has been shown to interact similarly with facial processing. It has been found that emotional sound can influence the affective face detection (de Gelder and Vroomen, 2000). The early interaction window between sound and facial processing started around 110 ms (Pourtois et al., 2000).

It is not difficult to understand that such cross-modal sensory information integration can happen at different stages. With evolution, such situations frequently occur in the natural environment, which forces human beings to develop optimal neural network processes for cross-modal information. These different successive ERP components provided the time-windows to observe the stages of cross-modal information interaction. For instance, emotional facial processing has been integrated with additional emotional cues like body gestures or voice information at an early stage. Through the four ERP components, one can observe the interaction effect and estimate the stage (e.g., initial automatic stage or high level of cognitive stage) of cross-modal interaction. Moreover, with taste and vision, Xiao et al. (2014) has found that the gustatory stimuli may interact with food images at the LPP stage. Hence, with affective facial processing as the task performance, one can study temporal dynamic integrations of other sensory information and facial processes.

The additional sensory information of vision and sound has provided a similar interaction effect on affective face recognition. However, is it necessary for the gustatory system to contribute to the affective face detection system in a similar manner? This question is still puzzling. Though previous studies have researched the olfactory system and have found some interaction effect between smell and face detection (Pause et al., 2004), the interaction between taste and emotional face processing remains unresolved. Therefore, we applied two different tastes (sweet and acidic) as gustatory stimuli in our experiment. The participants were made to keep their mouth’s taste and choose target faces with the distractor information (another face parallel on the screen). The four ERP components were analyzed to note if any interaction effect exists between taste and emotional face search. If yes, at which stage does it happen? How can sweet and acidic tastes influence the affective facial processing? All these questions will be addressed in this study.

## Materials and Methods

### Participants

G^∗^Power version 3.1 (Faul et al., 2007) is used to compute the required sample size before the experiment. With effect size f set at 0.25, and alpha set at 0.05 for two-tailed tests, a needed sample size of 23 gave us at least a 95% chance of observing a similar effect size. In our experiment, a total of 30 students from Liaoning Normal University (LNU) were selected, including 13 males and 17 females. The selection of a larger sample than the required size enabled us to reach a higher statistical power. The average age of the participants is 20.62 ± 1.34 (from 19 to 23) years old. They are all right-handed, with normal or corrected vision, no color blindness or color weakness, and no taste or smell related diseases. All the participants had to provide written informed consent, and the study was approved by the Institute Ethics Committee (IEC) of LNU. Each participant was paid for their participation.

### Stimuli

Thirty-six affective face images were selected from the Chinese Facial Affective Picture System (CFAPS) (Bai et al., 2005). (The pictures are available *via* the link^1^). Half of them were male faces and the other half were female faces. There were three types of emotional faces: positive, neutral, and negative. Each type contains 12 face images. With the emotional face search task, two face images were displayed parallelly on the screen (left and right), which allowed around 10° × 9° of visual angle. One emotional face was taken as a target emotional face (either positive or negative); the other distracting image was a neutral or opposite emotional face. The gustatory stimuli were vitamin C tablets (1,000 mg per tablet, Northeast Pharmaceutical Group Shenyang First Pharmaceutical Co., Ltd.) and sugar crystals (2,000 mg per piece, Hebei Yizhilian Co., Ltd.). The tablets or crystals were placed on the participant’s tongue during the experiment (Xiao et al., 2011).

### Experimental Procedure

After signing a consent form, participants were made to sit in front of a computer screen (Lenovo’s 23-inch LCD, screen refresh rate 60 Hz, resolution 1,680 × 1,080 pixels) in a sound-proof chamber and were fitted with a 64-channel ANT equipment (ANT Neuro) electrode cap. All electrodes were positioned according to the international 10–20 system (Binnie et al., 1982) and referenced to online CPz (central cortex) during the recording. The sampling rate of signal recording was 500 Hz. The electrodes M1 and M2 were placed on the left and right mastoid, respectively. An EOG (electrooculogram) was recorded from the electrodes placed both above and below each eye. Electrode impedance was maintained below 5 kΩ and a 0.01–100 Hz band-pass filter.

Before the formal experiment, the participants were trained in the practice section for as long as required. They learned to breathe through their nose, keep their bodies as still as possible, and fix their eyes on the monitor and avoid blinking or moving their eyes or mouth while performing the task.

The experiment was based on the two factors within the subject design. The two variables of emotional faces and taste were manipulated. The emotional faces had four levels: positive (neutral), positive (negative), negative (neutral), and negative (positive). The tastes had two levels: sweet or acidic. Thus, there were a total eight conditions of different stimuli combination (Table 1): Positive face (target) + Neutral face (distractor) + Sweet taste (PoNuS); Positive face (target) + Negative face (distractor) + Sweet taste (PoNgS); Negative face (target) + Neutral face (distractor) + Sweet taste (NgNuS); Negative face (target) + Positive face (distractor) + Sweet taste (NgPoS); Positive face (target) + Neutral face (distractor) + Acidic taste (PoNuA); Positive face (target) + Negative face (distractor) + Acidic taste (PoNgA); Negative face (target) + Neutral face (distractor) + Acidic taste (NgNuA); Negative face (target) + Positive face (distractor) + Acidic taste (NgPoA). To eliminate any gender interference, both the target and the distractor faces chosen were of the same gender.

**TABLE 1:** Eight conditions of different stimuli and the corresponding abbreviation.

Abbreviation	Target face	Distractor face	Taste
PoNuS	Positive	Neutral	Sweet
PoNgS	Positive	Negative	Sweet
NgNuS	Negative	Neutral	Sweet
NgPoS	Negative	Positive	Sweet
PoNuA	Positive	Neutral	Acidic
PoNgA	Positive	Negative	Acidic
NgNuA	Negative	Neutral	Acidic
NgPoA	Negative	Positive	Acidic

**FIGURE 1:**
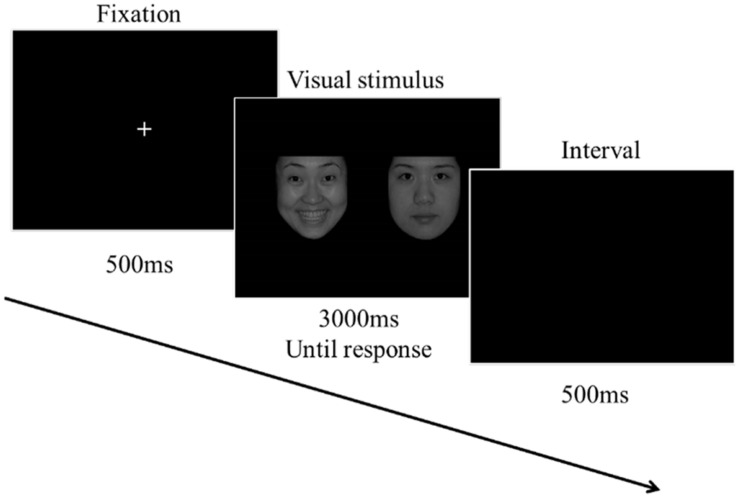
Schematic diagram of the experimental process.

The procedure is depicted in Figure 1. The experiment started with a white “+” fixation on the center of a black screen for 500 ms. After that, two emotional faces appeared. Participants needed to search the target face, then press the keyboard (“1” if the target is on the left and “2” if it is on the right) as quickly as possible. The face images would disappear as soon as the participants responded, and if the participants did not respond at all, the images would disappear after 3,000 ms. After every trial, the screen turned black for 500 ms as an interval before the next trial. The whole experiment included eight blocks and a total of 1,152 trials (each block 144 trials). The target face (e.g., Positive face) was fixed with one of these blocks. Half of the participants started responding to the block with a sweet taste, and the other half with an acidic taste. After every trial, the screen turned black for 500 ms as an interval before the next trial. Within each block, the gender and the side (left or right) of the target face were balanced, and the visual stimuli (PoNu, PoNg, NgNu, and NgPo) was randomly displayed. The participants had to rinse their mouth three times with purified water to remove the taste, and then rest for 120 s before the next block. The participants switched the taste materials (sweet and acidic) in the successive blocks. The taste material (a sugar crystal or a Vit C Tablet) remained on the tongue, and the sweet and acidic taste could last for the whole block (less than 5 min). (A pilot study has tested the change in the taste intensity of the same material over a prolonged time. It showed that the taste remained distinctly for 5 min. For the data, please refer to the Supplementary Material).

### Data Collection and Analysis

Brain Vision Analyzer 2.0 software (Brain Products GmbH, Germany) was used for data processing and EEG filtering (high pass as 0.01 Hz, a low pass is 30 Hz). The data were segmented into 1,000 ms intervals (200 ms pre-stimulus and 1,500 ms post-stimulus), and corrected to the 200 ms pre-stimulus baseline. Trials with correct behavioral responses were selected for further ERP analysis. Artifacts due to eye movements were corrected *via* the ocular correction software. Based on previous research (Schupp et al., 2004; Luo et al., 2010) and the plot map of this experiment, the four ERP components’ average amplitudes P1, N170, EPN, and LPP were selected for the analysis. For the P1 component, the recordings from the eight electrodes were chosen, Oz, O1, O2, POz, PO3, PO4, PO7, and PO8; the time window was 120–180 ms. For N170 and EPN components, P7, P8, PO7, and PO8 electrodes were selected as signal analysis; the time windows were 160–190 and 230–290 ms, respectively. Six electrodes of P3, P4, POz, Cp3, Cp4, and Cpz were used for LPP component; the time window was 450–550 ms. For the average grand potentials of each ERP component, please refer to the Supplementary Material. All data analyses were done using SPSS 21.0. Repeated measures analysis of variance (ANOVA) was adopted for each dependent variable, and to further analyze the significant interaction effects, simple effect tests (*t*-tests) with Bonferroni correction were performed. (The recorded EEG data are assessable *via* the link^2^).

## Results

### Behavior Results

**FIGURE 2:**
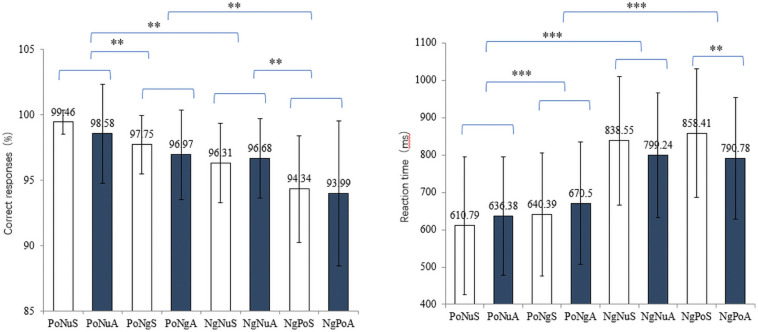
The left and right diagrams demonstrate the correct response ratios and the reaction time of different stimuli conditions. X-axis depicts the eight conditions: PoNuS, PoNuA, PoNgS, PoNgA, NgNuS, NgNuA, NgPoS, and NgPoA. ** and *** represent *p* < 0.01 and *p* < 0.001, respectively.

A 4 (emotional faces: PoNu, PoNg, NgNu, and NgPo) × 2 (taste: sweet and acidic) repeated measures ANOVA was used to test the main effects and interaction effect of correct response ratios. The main effect of emotional faces has been observed [*F*(3, 87) = 25.197, *p* < 0.001, η_*p*_^2^ = 0.448]. *Post hoc* tests revealed the correct response ratios of different visual combinations: PoNu (99.02% ± 2.80%) > PoNg (97.36% ± 2.93%) > NgNu (96.06% ± 3.04%) > NgPo (94.17% ± 4.86%) (Figure 2 left). There was no significant main effect of taste [*F*(1, 29) = 1.022, *p* = 0.320, η*_*p*_^2^* = 0.032]. There was no significant interaction effect between vision and taste [*F*(3, 87) = 0.668, *p* = 0.565, η*_*p*_^2^* = 0.021].

The reaction time (RT) of trials with the correct response was selected and further examined (Figure 2 right). Repeated ANOVA showed that the main effect of emotional faces was significant [*F*(3, 87) = 109.697, *p* < 0.001, η*_*p*_^2^* = 0.780]. *Post hoc* tests revealed the RT with different visual stimuli: PoNu (623.59 ± 172.91 ms) < PoNg (655.45 ± 164.74 ms) < NgNu (818.90 ± 170.57 ms) < NgPo (824.59 ± 170.28 ms). There was no significant main effect of taste [*F*(1, 29) = 1.946, *p* = 0.173, η*_*p*_^2^* = 0.059]. However, the notable interaction between vision and taste existed [*F*(3, 87) = 10.349, *p* < 0.001, η*_*p*_^2^* = 0.250]. A simple effect test with Bonferroni correction only revealed that the RT of NgPoA (790.78 ± 162.02 ms) was remarkably shorter than that of NgPoS [858.41 ± 171.63 ms, *t*(31) = −3.558, *p* < 0.01]. All other pairwise comparisons were not significant (*p*s > 0.05).

### ERP Components

#### P1 (120–180 ms)

**FIGURE 3:**
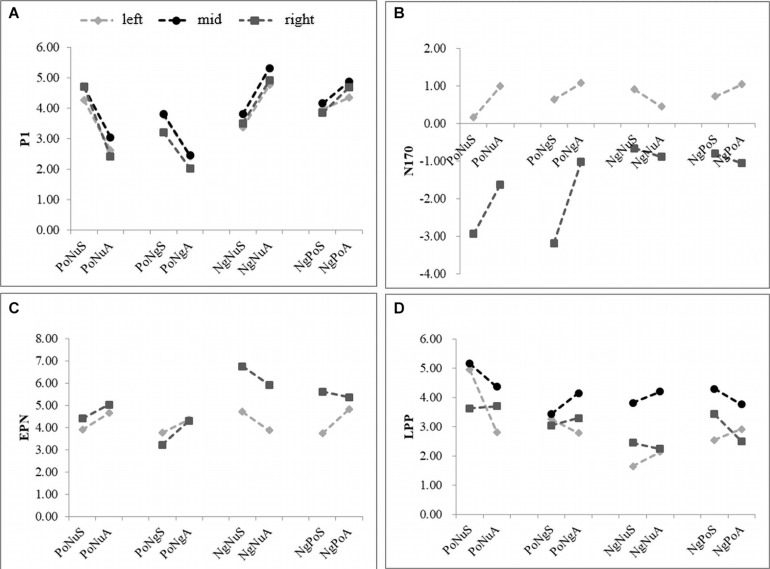
Panels **(A–D)** represent the average response amplitude of P1, N170, EPN, and LPP, respectively. X-axis represents the eight different stimuli combinations, and Y-axis represents the average amplitude in μv. The gray dots, black dots, and black squares represented the left, mid, and right brain responses, respectively.

A 3 (area: left, center and right hemisphere) × 4 (emotional faces: PoNu, PoNg, NgNu, and NgPo) × 2 (taste: sweet and acidic) repeated-measures ANOVA was used to analyze P1 (Figure 3A and Table 2). The main effect of emotional faces was significant [*F*(3, 87) = 36.491, *p* < 0.001, η_*p*_^2^ = 0.557]. The amplitude of the target negative faces (NgNu: 4.27 ± 0.57; NgPo: 4.31 ± 0.58) was significantly larger than that of the target positive faces (PoNu: 3.62 ± 0.58; PoNg: 2.94 ± 0.51) (*p* < 0.001). There was no main effect of taste [*F*(1, 29) = 1.294, *p* = 0.265, η_*p*_^2^ = 0.043] and brain areas [*F*(2, 58) = 0.759, *p* = 0.435, η_*p*_^2^ = 0.025].

**TABLE 2:** Results of the analyses of variance for the ERP component P1, N170, EPN, and LPP.

	Main effect			Interaction effect			
	Area*	Vision	Taste	Area × vision	Area × taste	Vision × taste	Area × vision × taste
P1	*F*(2,58) = 0.759 *p* = 0.435	*F*(3,87) = 36.491 *p* < 0.001	*F*(1,29) = 1.294*p* = 0.265	*F*(6,174) = 3.641*p* < 0.05	*F*(2,58) = 0.933 *p* = *0*.399	*F*(3,87) = 37.635 *p* < 0.001	*F*(6,174) = 2.372 *p* = 0.079
N170	*F*(1,29) = 6.604 *p* < 0.05	*F*(3,87) = 5.960 *p* < 0.01	*F*(1,29) = 5.830 *p* < 0.05	*F*(3,87) = 17.200 *p* < 0.001	*F*(1,29) = 2.671*p* = *0.*113	*F*(3,87) = 12.279 *p* < 0.001	*F*(3,87) = 4.723 *p* < 0.05,
EPN	*F*(1,29) = 1.559 *p* = 0.222	*F*(3,87) = 12.797 *p* < 0.001	*F*(1,29) = 1.869*p* = 0.182	*F*(3,87) = 26.937 *p* < 0.01	*F*(1,29) = 0.338 *p* = 0.565	*F*(3,87) = 6.227 *p* < 0.01	*F*(3,87) = 3.927 *p* < 0.05
LPP	*F*(2,58) = 12.969 *p* < 0.001	*F*(3,87) = 15.391 *p* < 0.001	*F*(1,29) = 1.893*p* = 0.179	*F*(6,174) = 4.836 *p* < 0.01	*F*(2,58) = 2.843 *p* = 0.072	*F*(3,87) = 1.557 *p* = 0.212	*F*(6,174) = 6.388 *p* < 0.01

*Area means the brain area.

The interaction between taste and vision was substantial [*F*(3, 87) = 37.635, *p* < 0.001, *η_*p*_^2^* = 0.565]. A simple effect test was conducted to show that PoNuS (4.56 ± 0.56) stirred larger responses than PoNuA (2.68 ± 0.62) (*p* < 0.001) (Figure 3A). PoNgS (3.59 ± 0.50) instigated larger responses than PoNgA (2.29 ± 0.54) (*p* < 0.001). NgNuA (5.00 ± 0.68) induced larger responses than NgNuS (3.55 ± 0.50) (*p* < 0.01).

The interaction between brain area and vision was significant [*F*(6, 174) = 3.641, *p* < 0.05, *η_*p*_^2^* = 0.112]. A simple effect test showed, in all brain areas, NgNu and NgPo initiated stronger responses than PoNu and PoNg, respectively (*p* < 0.001; *p* < 0.01). In the mid and right brain areas, responses of PoNu are larger than those of PoNg (*p* < 0.001).

There is no interaction between area and taste [*F*(2, 58) = 0.933, *p* = 0.399, η_*p*_^2^ = 0.031], nor among area, taste, and vision [*F*(6, 174) = 2.374, *p* = 0.079, η_*p*_^2^ = 0.076].

#### N170 (160–190 ms)

Similar analysis has been applied (Table 2). The main effect has been observed in brain areas [*F*(1, 29) = 6.604, *p* < 0.05, *η_*p*_^2^* = 0.185]. The right hemisphere (−1.53 ± 0.98) responded stronger than the left hemisphere (0.75 ± 0.40). The emotional faces have shown the main effect [*F*(3, 87) = 5.960, *p* < 0.01, *η_*p*_^2^* = 0.170]. PoNu (−0.86 ± 0.62) induced larger amplitudes than NgNu (−0.05 ± 0.54) (*p* < 0.01). The taste had a main effect as well [*F*(1, 29) = 5.830, *p* < 0.05, *η_*p*_^2^* = 0.167]. Sweet (−0.65 ± 0.63) caused stronger responses than Acidic (−0.13 ± 0.59).

The interaction was significant between vision and taste [*F*(3, 87) = 12.279, *p* < 0.001, *η_*p*_^2^* = 0.297]. A simple test has demonstrated that PoNuS (−1.39 ± 0.65) has led to larger responses than PoNusA (−0.32 ± 0.62) (*p* < 0.01). PoNgS (−1.28 ± 0.74) induced larger responses than PoNgA (0.02 ± 0.75) (*p* < 0.001).

The interaction effect between the area and vision was commendable [*F*(3, 87) = 17.200, *p* < 0.001, *η_*p*_^2^* = 0.372]. A simple test showed that on the right hemisphere PoNu (−2.29 ± 0.97) influenced larger responses than PoNg (−0.78 ± 0.89) (*p* < 0.001).

No noticeable interaction effect was shown between area and taste [*F*(1, 29) = 2.671, *p* = 0.113, and *η_*p*_^2^* = 0.084].

The interaction among vision, taste, and brain areas was significant [*F*(3, 87) = 4.723, *p* < 0.05, and *η_*p*_^2^* = 0.140]. A simple effect test was conducted to show that PoNuS (0.16 ± 0.41) induced stronger negative response than PoNuA (0.99 ± 0.54) (*p* < 0.01) on the left hemisphere, whereas NgNuA (0.45 ± 0.35) induced stronger negative response than NgNuS (0.91 ± 0.39) (*p* < 0.01). NgNuA (0.45 ± 0.35) induced stronger negative response than NgNuS (0.91 ± 0.39) (*p* < 0.01). On the right hemisphere, on the other hand, PoNuS (−2.94 ± 1.12) induced a stronger negative response than PoNuA (−1.63 ± 0.86) (*p* < 0.05) and PoNgS (−3.19 ± 1.21) induced a stronger negative response than PoNgA (−1.03 ± 1.06) (*p* < 0.001).

#### EPN (250–290 ms)

A similar analysis has been applied and it was found that the main effect of the emotional faces was evident [*F*(3, 87) = 12.797, *p* < 0.001, η_*p*_^2^ = 0.306]. PoNu (4.49 ± 0.84) induced more negative responses than NgNu (5.31 ± 0.75) (*p* < 0.01), while PoNg (3.91 ± 0.88) induced more negative responses than NgPo (4.88 ± 0.77) (*p* < 0.05) and PoNg induced more negative responses than PoNu (*p* < 0.05). There was no noteworthy effect of the brain areas [*F*(1, 29) = 1.559, *p* = 0.222, η_*p*_^2^ = 0.051], nor was there any significant impact from the taste [*F*(1, 29) = 1.869, *p* = 0.182, η_*p*_^2^ = 0.061].

The interaction between taste and emotional faces was significant [*F*(3, 87) = 6.227, *p* < 0.01, η*_*p*_^2^* = 0.178]. A simple effect test was conducted which showed that PoNgS (4.15 ± 0.89) induced more negative responses than PoNuA (4.84 ± 0.81) (*p* < 0.05); PoNgS (3.50 ± 0.87) induced more negative responses than PoNgA (4.33 ± 0.91) (*p* < 0.01) and NgNuA (4.89 ± 0.87) induced more negative responses than NgNuS (5.73 ± 0.65) (*p* < 0.05). Significant interaction was observed between emotional faces and brain areas [*F*(3, 87) = 26.937, *p* < 0.01, and η*_*p*_^2^* = 0.482]. A simple test showed that, on the right hemisphere, PoNu (4.71 ± 0.90) induced more negative responses than NgNu (6.33 ± 0.83) (*p* < 0.001); PoNg (3.76 ± 0.85) induced more negative responses than NgPo (5.45 ± 0.83) (*p* < 0.01). PoNg induced more negative responses than PoNu (*p* < 0.01) and NgPo induced more negative responses than NgNu (*p* < 0.01). No significant interaction was seen between brain areas and taste [*F*(1, 29) = 0.338, *p* = 0.565, and η*_*p*_^2^* = 0.012].

The interaction among vision, taste, and brain areas was noteworthy [*F*(3, 87) = 3.927, *p* < 0.05, and η*_*p*_^2^* = 0.119]. A simple test has shown that on the left hemisphere, PoNuS (3.90 ± 0.92) induced more negative responses than PoNuA (4.65 ± 0.92) (*p* < 0.01) and PoNgS (3.77 ± 1.01) induced more negative responses than PoNgA (4.35 ± 1.09) (*p* < 0.05). Whereas on the right hemisphere, PoNuS (3.22 ± 0.93) induced more negative responses than PoNuA (4.30 ± 0.84) (*p* < 0.05) and PoNgS (5.92 ± 0.92) induced more negative responses than PoNgA (6.75 ± 0.76) (*p* < 0.05).

#### LPP (450–550 ms)

The main effect of emotional faces was significant [*F*(3, 8) = 15.391, *p* < 0.001, and η_*p*_^2^ = 0.347]. PoNu (4.10 ± 0.37) induced more positive responses than NgNu (2.74 ± 0.28) (*p* < 0.001); on the other hand, PoNu induced more positive responses than PoNg (3.32 ± 0.34) (*p* < 0.01). There was a significant main effect of brain areas [*F*(2, 58) = 12.969, *p* < 0.001, and η*_*p*_^2^* = 0.309]. The average responses on the right brain (4.14 ± 0.44) were more positive than those of the mid brain (3.03 ± 0.32) and the left brain (2.88 ± 0.28) (*p* < 0.01). There was no main effect from taste [*F*(1, 29) = 1.893, *p* = 0.179, and η*_*p*_^2^* = 0.061].

There was no interaction between emotional faces and taste [*F*(3, 87) = 1.557, *p* = 0.212, and η*_*p*_^2^* = 0.051], or between brain areas and taste [*F*(2, 58) = 2.843, *p* = 0.072, and η*_*p*_^2^* = 0.089]. Significant interaction was observed between emotional faces and brain areas [*F*(6, 174) = 4.836, *p* < 0.01, and η*_*p*_^2^* = 0.143]. A simple effect test was conducted and it showed that PoNu (3.88 ± 0.39) induced more positive responses than NgNu (1.89 ± 0.19) in the whole brain (*p* < 0.001 for the left and mid brain; *p* < 0.05 for the right brain). Additionally, PoNu initiated more positive responses than PoNg (3.02 ± 0.34) on the left brain (*p* < 0.001). The interaction among vision, taste, and brain areas was significant [*F*(6, 174) = 6.388, *p* < 0.01, and η*_*p*_^2^* = 0.181]. This simple test also showed that on the left brain, PoNuS (4.95 ± 0.74) induced more positive responses than PoNuA (2.81 ± 0.29) (*p* < 0.05).

## Discussion

Our results have shown that gustatory stimuli can influence affective facial processing at behavioral and neural levels. Behavior data has shown that while detecting a negative target face with a positive face as a distractor, the participants perform the task faster with the acidic taste than with the sweet taste. With temporal dynamic ERP analysis, the significant interaction effects between the emotional face and the taste have been observed with P1, N170, and EPN.

In previous literature, multiple sensory stimuli have been applied as the congruent or incongruent emotional background to observe the cross-modal interaction for emotion detection at behavioral and physiological levels (Klasen et al., 2012). Usually, the congruent stimuli combination can induce faster responses than the incongruent ones. For instance, the subjects responded considerably faster when the emotional valence of the sound and the facial expression were congruent than during the incongruent conditions (Föcker et al., 2011; Müller et al., 2011). However, with an olfactory input, Seubert et al. (2010) showed that both positive and negative odors improve the subjects’ recognition of disgusted faces’ speed and accuracy. Our behavior results here are consistent with some of the previous behavioral observations. On one side, participants searched the target of negative faces faster when they had the acidic taste (congruent) than that when they had the sweet taste (incongruent) in the mouth. On the other side, no difference in reaction time was observed while searching positive faces with a sweet or acidic taste in the mouth. It has been reported that the brain response pattern can be asymmetric with positive and negative stimuli (Alves et al., 2009). Particularly, the similar behavior paradigm can lead to inconsistent response patterns, because too many system interferences cause insensitivity. Thereby, the electrophysiological level’s neural response patterns should be more sensitive and robust to observe the interaction effect of cross-modal sensory integration.

In previous ERP studies, the main observation of cross-modal interaction was focused on the early, mid, and late time windows of facial processing (Pourtois et al., 2000). The emotionally congruent stimuli induce larger ERP components than the emotionally incongruent stimuli (Righart and de Gelder, 2006; Hietanen and Astikainen, 2013). When the emotion expressed by scenes and facial expressions was constant, it stimulated greater N170 amplitude (Hietanen and Astikainen, 2013). Righart and de Gelder (2006) had placed fear and neutral face pictures in fear and neutral natural scene pictures and asked the subjects to judge whether the face was upright or inverted. They found that fear scenes’ faces stimulated a larger N170, and fear faces stimulated the largest N170. It was comprehensible that the congruent affective stimuli from other sensory modalities may improve emotion signal detection performance. With the facial process at the early stage (P1 and N170), the facial information is processed automatically, particularly sensitive to negative facial expression (Luo et al., 2010). Like body gesture and voice, other sensory cues, too, can automatically be integrated and interacted with at an early stage (Pourtois et al., 2000). In our study, the early-stage interaction was observed with P1 (120–180 ms) and N170 (160–190 ms). Hence, our data has extended from the previous understanding of cross-modal sensory interaction and suggested that taste, similar to other sensory inputs, can modify the affective face processing at an early stage of automatic sensory processing.

Both EPN and LPP have been taken as the prototypical emotional-related ERP comments. The EPN has implicated early tagging and prioritized processing (Schupp et al., 2004). The LPP has been suggested to indicate the reflection of high-level evaluation, affective labeling, and episodic memory encoding (Schupp et al., 2006). It has been demonstrated that the visual scenes, hand gestures, or videos can modulate both components with emotional facial processing (Schupp et al., 2006; Olofsson et al., 2008; Flaisch et al., 2009; Wiggert et al., 2015). With limited studies of vision and taste interaction, Schienle et al. (2017) found that the bitter aftertaste influences facial processing with P200 and P300. Xiao et al. (2011, 2014) have demonstrated that the congruent taste and food name or food item images provoke larger waves than the incongruent ones in the time window of around 400–600 ms. However, we did not observe any interaction effect with LPP in our experiments. In another parallel study, we observed the taste and face recognition interaction effect with LPP (manuscript submitted). In that experiment, the participant needed to identify the emotion of a single face displayed on the screen. The reason why we did not observe the interaction effect with LPP might be the different attention orientation. In that experiment (manuscript submitted), the subject only focused on a single emotional face. Whereas in the current study, the participants were asked to identify the face that matched the target emotion, among the parallel faces, shown on the screen, and then press the keyboard to present the right or left side with the target face accordingly. Their attention was not only focused on the affective face but also the spatial information (where the face is right or left). At the late stage of facial processing (LPP), where the high-level cortex is involved, the brain has parallel pathways. The bottom-up information is from visual and gustatory sensations, whereas the top-down control is from the higher cortex to evaluate and avoid distracting face information. During this stage, the face search task involves complex neural networks from memory, emotion, executives, and so on. Such variant attributes might lead to intriguing modifications, so we did not observe the interaction effect in our experiments.

It is important to note that the current study has some limitations. The taste stimuli were kept in the mouth throughout the task performance. The subject could not avoid taste adaptation effect, although our pilot study showed that the taste could remain within each block task. It has been shown that visual perception can be modulated by even time interval adaptation (Zhang et al., 2012). Thus, it should be more sensitive and precise to apply timely-locked taste stimuli in future research. Moreover, we found that gustatory information interacts with affective facial processing at different processing stages, the early and mid-stage of the facial process (P1, N170, and EPN). The interaction effects of this study were consistent with our previous observation (unpublished data from our laboratory with visual perception influenced by gustatory stimuli), but not consistent with Xiao et al. (2011), particularly with LPP time period. It indicates that the cross-modal interaction effect may be more task sensitive and relevant. More precisely, it means the late ERP processing could be more involved with particular task-related brain areas. Therefore, in future, different task paradigms could be applied in the cross-modal studies.

Taken together, our finding has provided the behavioral and electrophysiological evidence that sweet and acidic tastes could interact with the emotional face search process. We expect that the cross-modal interaction patterns observed at the electrophysiological level are more sensitive and robust than the behavior level’s response pattern. However, we are also aware of the constraints on generality (Simons et al., 2017) mentioned above. We have no reason to believe that the results depend on other characteristics of the participants, materials, or context.

## Data Availability Statement

The datasets presented in this study can be found in online repositories. The names of the repository/repositories and accession number(s) can be found in the article/Supplementary Material.

## Ethics Statement

The studies involving human participants were reviewed and approved by Institute Ethics Committee of Liaoning Normal University. The patients/participants provided their written informed consent to participate in this study. Written informed consent was obtained from the individual(s), and minor(s)’ legal guardian/next of kin, for the publication of any potentially identifiable images or data included in this article.

## Author Contributions

PL and JJ designed the experiment. JJ and JC performed the experiments. JJ, LW, and PL performed the data analysis. PL wrote the manuscript. LW and JC improved the manuscript. All authors contributed to the article and approved the submitted version.

## Conflict of Interest

The authors declare that the research was conducted in the absence of any commercial or financial relationships that could be construed as a potential conflict of interest.

## Footnotes

1 https://doi.org/10.6084/m9.figshare.13887428.v1

2 https://figshare.com/articles/dataset/EEG_data_rar/13888553
