# Supplementary figures and images for: Affective Face Processing Modified by Different Tastes
Source: Front Psychol. 2021 Mar 12;12:644704. doi: 10.3389/fpsyg.2021.644704 (PMC8006344; doi:10.3389/fpsyg.2021.644704)

# P1

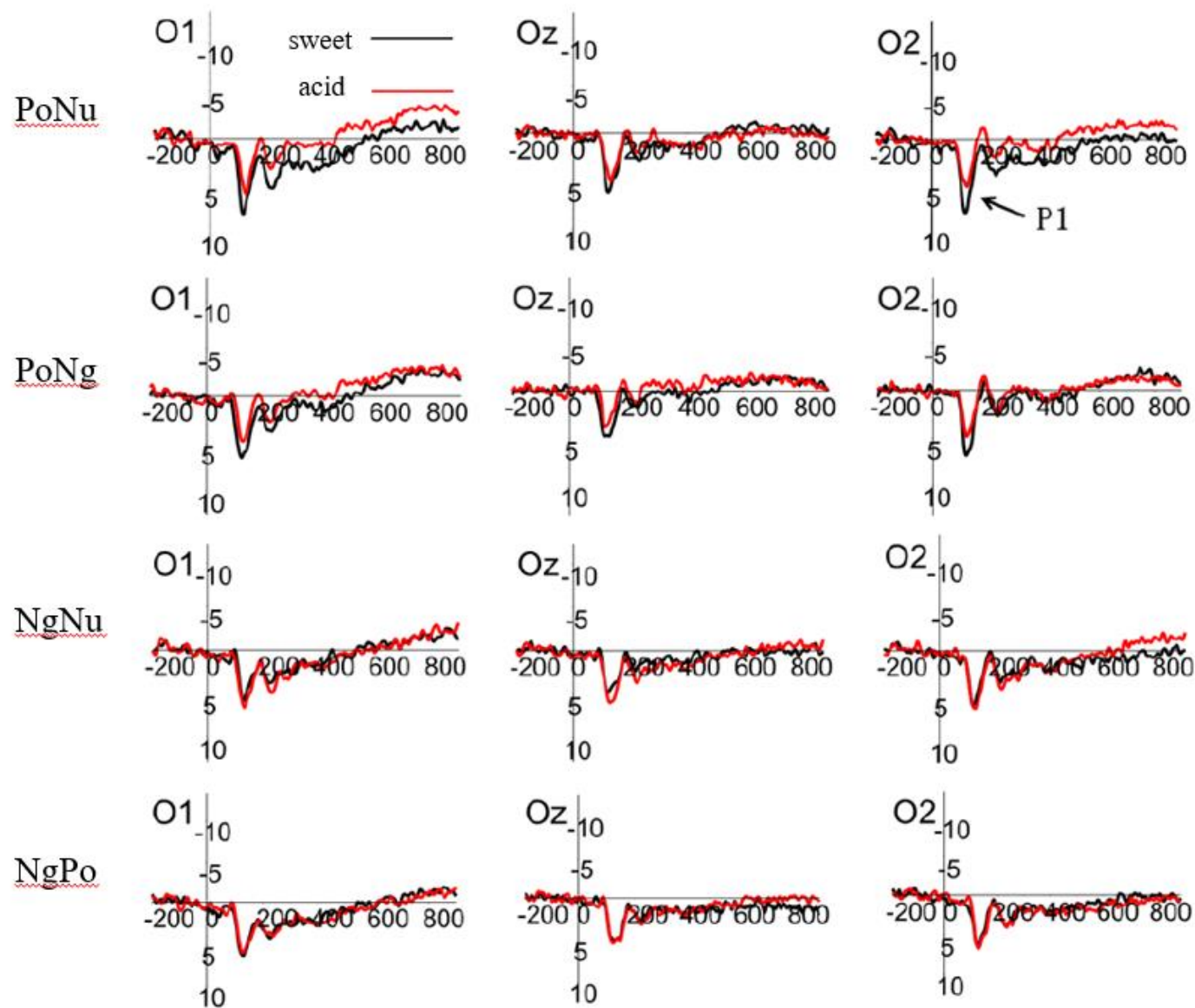

N170  
EPN

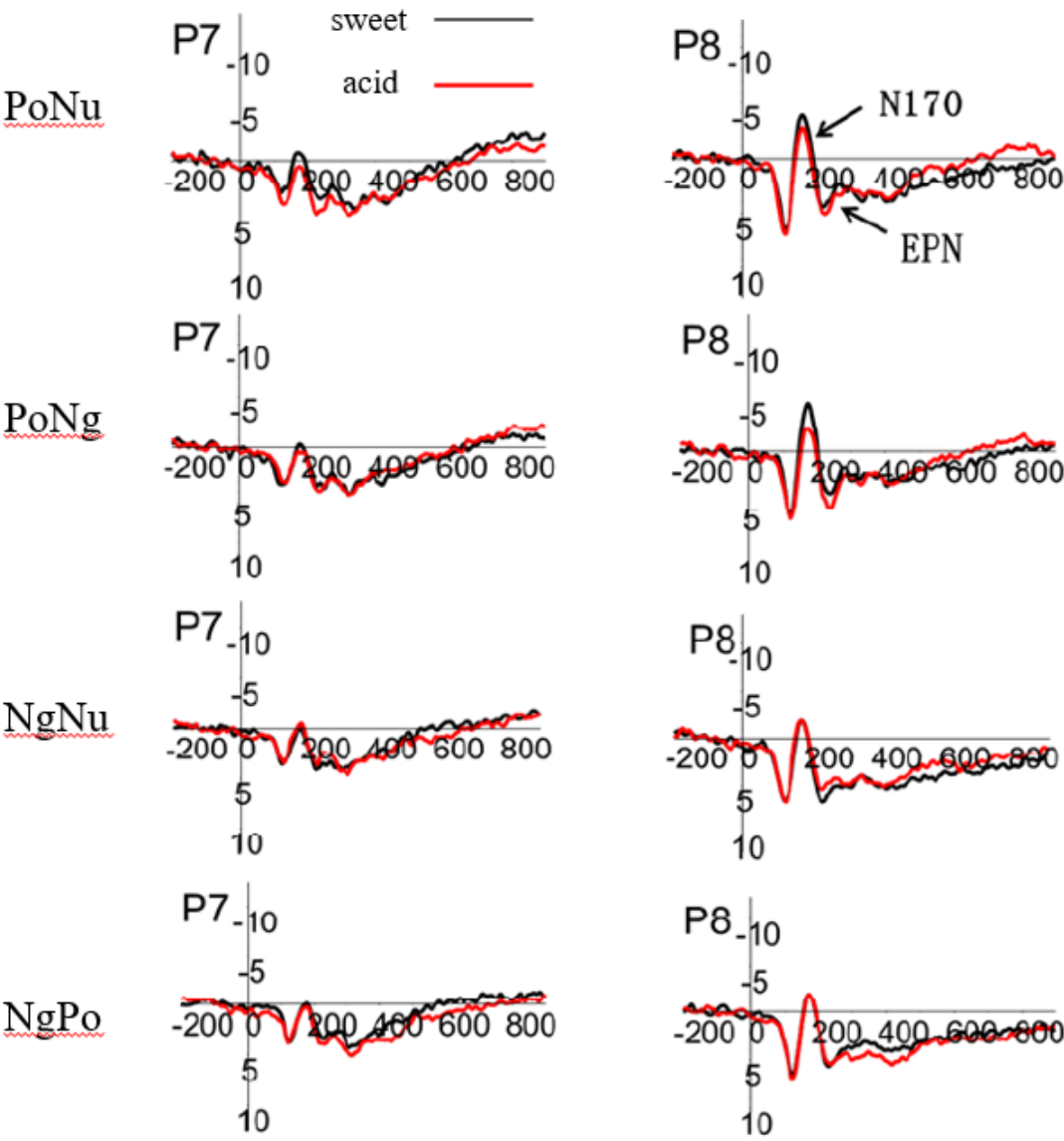

# LPP

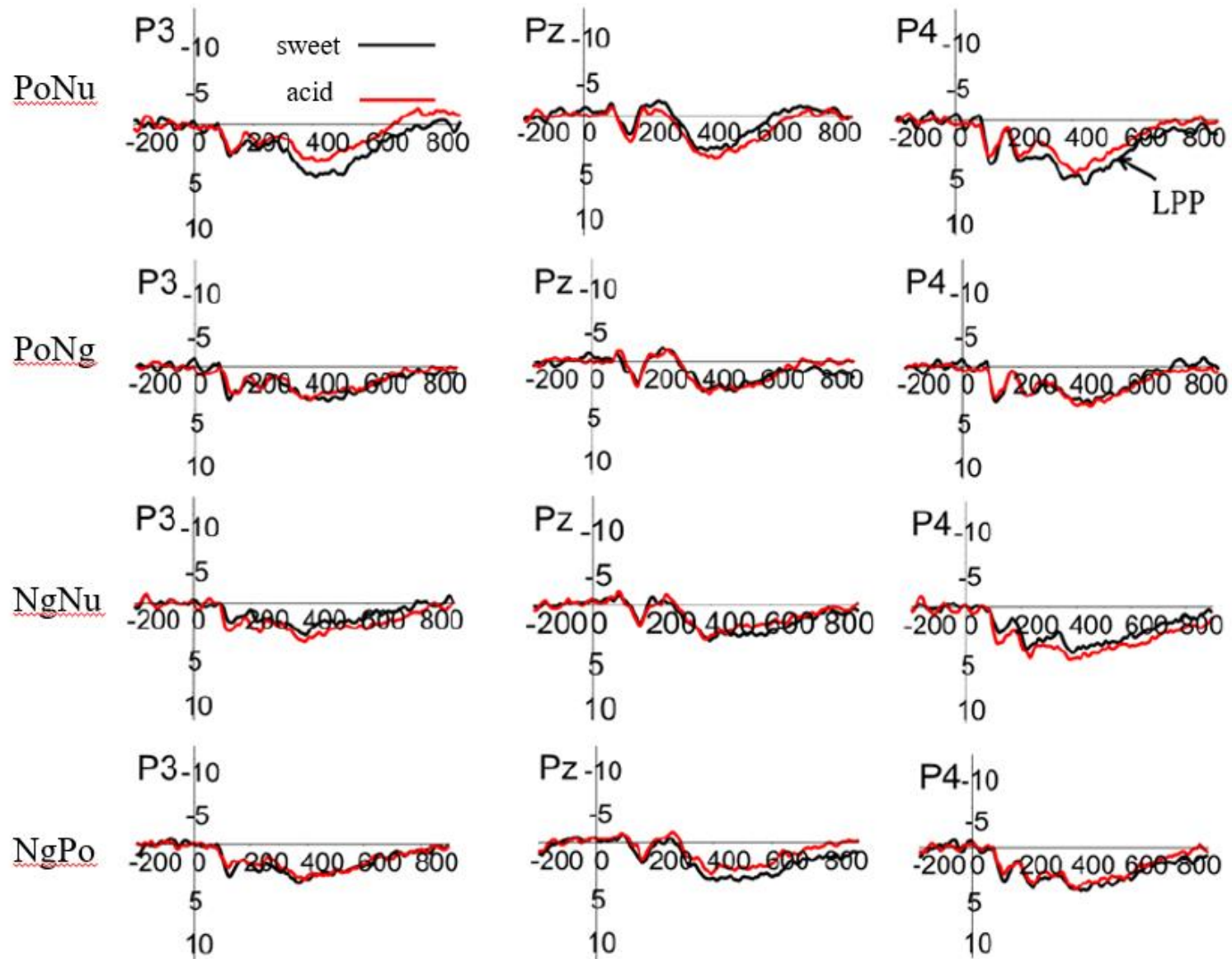

Supplement: Supplementary file 3 [file Data_Sheet_3.pdf]
